# Supplementary material for: Venomix: a simple bioinformatic pipeline for identifying and characterizing toxin gene candidates from transcriptomic data
Source: PeerJ. 2018 Jul 31;6:e5361. doi: 10.7717/peerj.5361 (PMC6074769; doi:10.7717/peerj.5361)
Supplement: Supplemental Information 3 [file peerj-06-5361-s003.gz › FinalOutput_E-6/Hyaluronidase_conohyal-Cn1_1/finaltree.pdf]

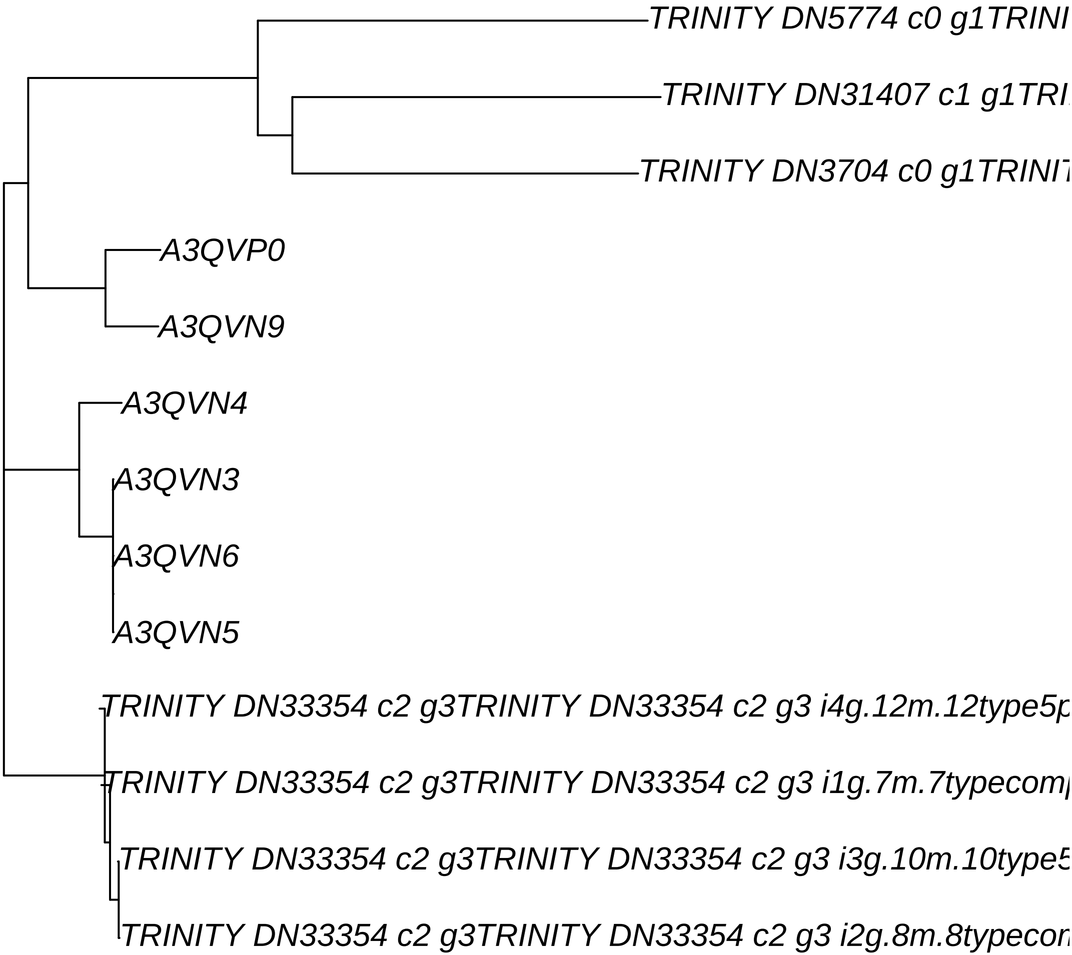

TRINITY DN5774 c0 g1TRINI
TRINITY DN31407 c1 g1TRI
TRINITY DN3704 c0 g1TRINIT
A3QVP0
A3QVN9
A3QVN4
A3QVN3
A3QVN6
A3QVN5
TRINITY DN33354 c2 g3TRINITY DN33354 c2 g3 i4g.12m.12type5p
TRINITY DN33354 c2 g3TRINITY DN33354 c2 g3 i1g.7m.7typecomp
TRINITY DN33354 c2 g3TRINITY DN33354 c2 g3 i3g.10m.10type5
TRINITY DN33354 c2 g3TRINITY DN33354 c2 g3 i2g.8m.8typecom
